# Mesenchymal Stem Cells Antagonize IFN-Induced Proinflammatory Changes and Growth Inhibition Effects via Wnt/β-Catenin and JAK/STAT Pathway in Human Outer Root Sheath Cells and Hair Follicles

**DOI:** 10.3390/ijms22094581

**Published:** 2021-04-27

**Authors:** Yu-Jin Lee, Song-Hee Park, Hye-Ree Park, Young Lee, Hoon Kang, Jung-Eun Kim

**Affiliations:** 1 Department of Dermatology, Eunpyeong St. Mary’s Hospital, College of Medicine, The Catholic University of Korea, Seoul 03312, Korea; cindyeujine1@naver.com (Y.-J.L.); saccharide@hanmail.net (S.-H.P.); 5953hari@naver.com (H.-R.P.); johnkang@catholic.ac.kr (H.K.); 2 Department of Dermatology, School of Medicine, Chungnam National University, Daejeon 35015, Korea; resina20@cnu.ac.kr

**Keywords:** mesenchymal stem cell therapy, Wnt/β-catenin pathway, JAK/STAT pathway, hair follicle, outer root sheath cells

## Abstract

Mesenchymal stem cell therapy (MSCT) has been shown to be a new therapeutic option for treating alopecia areata (AA). Outer root sheath cells (ORSCs) play key roles in maintaining the hair follicle structure and supporting the bulge area. In human ORSCs (hORSCs), the mechanism for this process has not been extensively studied. In this study, we aimed to examine the influence of human hematopoietic mesenchymal stem cells (hHMSCs) in the hORSCs in vitro model of AA and determine the mechanisms controlling efficacy. Interferon-gamma (IFN-γ) pretreatment was used to induce an in vitro model of AA in hORSCs. The effect of MSCT on the viability and migration of hORSCs was examined using co-cultures, the MTT assay, and migration assays. We investigated the expression of molecules related to the Wnt/β-catenin pathway, JAK/STAT pathway, and growth factors in hHMSC-treated hORSCs by reverse transcription-polymerase chain reaction (RT-PCR) and Western blot analyses. hHMSCs increased hORSC viability and migration when they were co-cultured. hHMSCs reverted IFN-γ-induced expression—including NLRP3, ASC, caspase-1, CXCL-9 through 11, IL-1β, and IL-15—and upregulated several growth factors and hair stem cell markers. hHMSCs activated several molecules in the Wnt/β-catenin signaling pathway, such as in the Wnt families, β-catenin, phosphorylated GSK-3β and cyclin D1, and suppressed the expression of DKK1 induced by IFN-γ in hORSCs. hHMSCs suppressed the phosphorylation of JAK1 to 3, STAT1, and STAT3 compared to the controls and IFN-γ-pretreated hORSCs. These results demonstrate that hHMSCs increased hORSC viability and migration in the in vitro AA model. Additionally, MSCT definitely stimulated anagen survival and hair growth in an HF organ culture model. MSCT appeared to be associated with the Wnt/β-catenin and JAK/STAT pathways in hORSCs.

## 1. Introduction

Alopecia areata (AA) is an autoimmune disease that targets hair follicles (HFs) during the anagen stage and is primarily caused by the disruption of immune privilege. [1,2]. AA is characterized by unexpected hair loss [3,4]. Even though AA is not a serious, life-threatening illness, it can have psychological consequences, including high levels of depression and anxiety [1,2]. However, the pathological mechanisms of chronic AA and alopecia totalis or alopecia universalis hair loss are not fully understood. Many patients with alopecia totalis and alopecia totalis show tolerance to conventional treatments [5].

According to the concept of pathology, the prevention or recovery of immune privilege disruption of HFs can ultimately lead to the more effective management of AA. Recently, research studies have reported that treatments using mesenchymal stem cells (MSCs) can be useful for treating AA. Mesenchymal stem cell therapy (MSCT) or stem cell therapy using umbilical cord blood-derived cells for AA has been reported in several refractory cases [6,7]. A recent study examined the effects of MSCT in AA mouse models and discovered the immunomodulatory effects of MSCT. MSCT inhibited the expression of interferon-gamma (IFN-γ), C-X-C motif chemokines (CXCL) 9 and 10, and T-cell infiltration around HFs in AA mice [8].

The mechanism by which MSCT is associated with improvement of hair growth and the immune environment is not yet well known. According to our previous study, MSCT reverted an AA-like environment in human dermal papillar cells (hDPCs) via the Wnt/β-catenin and Janus kinase (JAK)/signal transducers and activators of transcription protein (STAT) signaling pathways. In addition, various growth factors and cytokines associated with anagen re-entry induction were found to be enhanced by MSCT [9]. Activation of the Wnt/β-catenin and JAK/STAT signaling pathways is known to upregulate genes responsible for cell differentiation, proliferation, and anagen reentry in HFs [10,11]. MSCT was found to prolong anagen re-entry through activation of the Wnt/β-catenin and JAK/STAT signaling pathways [9].

HFs are composed of cells of multiple origins, are single independent mini-organs, and have a characteristic periodic hair cycle [12]. The hair starts anew by the interaction between hDPC and hORSC signals. Additionally, some hORSCs contain HF stem cells, which are important for the initiation of the hair cycle.

The hORSCs of HFs are multilayered tissues predominantly made up of undifferentiated keratinocytes [13]. hORSCs mainly comprise the hair shaft and the hair matrix. They play important roles in maintaining hDPCs and hair matrix cells [14,15,16,17]. They also contribute to the formation of HFs and the epidermis. hORSC apoptosis, which influences cell degeneration, the catagen phase of HFs, and upregulates the levels of necrosis and apoptosis in HFs, is associated with AA [18]. hORSCs also regulate inflammatory responses by expressing NLR family pyrin domain containing 3 (NLRP3) [19].

The effects of MSCT on hORSCs have not yet been studied. To study the therapeutic mechanism of MSCT in AA, we investigated the effect of MSCT on hORSCs, with or without pretreatment with IFN-γ. We examined the effect of MSCT on the viability and migration ability of hORSCs. We focused on the activation of the Wnt/β-catenin and JAK/STAT signaling pathways and changes associated with the expression of several cytokines and chemokines, inflammasomes, growth factors, hair stem cell markers, and migration in response to MSCT.

## 2. Results

### 2.1. Effect of hHMSC Treatment on the Cell Viability of hORSCs

**Figure 1:**
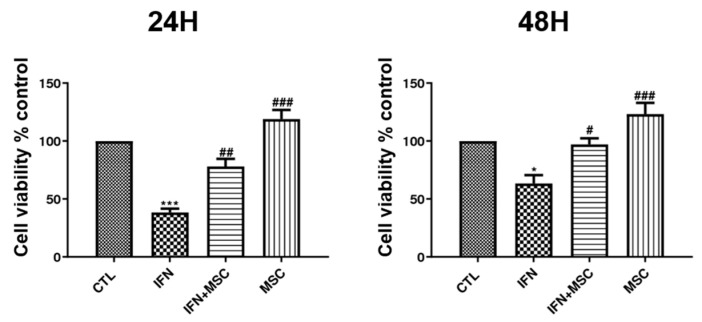
The effect of hHMSC treatment on the viability of hORSCs was evaluated by the MTT assay. Cell viability was detected in hORSCs (control), hORSCs treated with IFN-γ, hORSCs treated with IFN-γ and co-cultured with hHMSCs, and co-cultures of hORSCs and hHMSCs. Cell viability was increased in the hHMSC coculture. Error bars represent the mean ± SEM, n = 3. Statistically significant at * *p* < 0.05 and *** *p* < 0.001, compared to the control and # *p* < 0.05, ## *p* < 0.01, and ### *p* < 0.001 compared to the IFN-γ-treated group.

First, we examined whether MSCT could increase the cell viability of hORSCs. The concentration of hHMSCs used was 5 × 10^4^ cells/well, according to a previous study [9]. To determine time-dependent effects, hORSCs were co-cultured with hHMSCs for 24 h and 48 h. Cell viability was estimated by MTT assays (Figure 1). The results showed that treatment with hHMSCs enhanced hORSC viability at 24 h and 48 h. While IFN-γ significantly decreased the cell viability of hORSCs, when co-cultured with hHMSCs, hORSC viability increased up to ~130% compared to the controls. Furthermore, MSCT upregulated hORSC viability in the IFN-γ–pretreated group compared to the IFN-γ-only treated group. This tendency was similar to that seen when hHMSCs were treated for 24 h and 48 h. In this study, we used 24 h for hHMSC and hORSC co-cultures in all experiments.

### 2.2. Effect of hHMSC Treatment on NLRP3 Inflammasome Components

**Figure 2:**
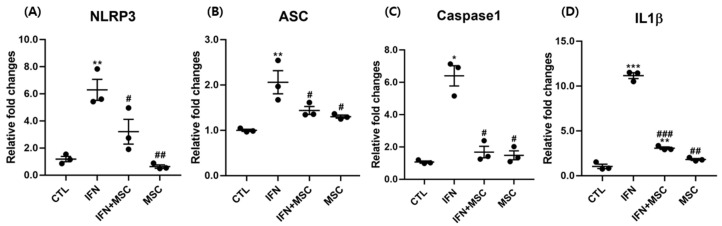
Analysis of gene expression by RT-PCR. The mRNA expression of (**A**) NLRP3, (**B**) ASC, (**C**) caspase-1, and (**D**) IL-1β in hORSCs. The NLRP3 inflammasome components were increased in the IFN-γ-treated group and decreased in the MSCT group. Error bars represent the mean ± SEM, n = 3. Statistically significant at * *p* < 0.05, ** *p* < 0.01, and *** *p* < 0.001 compared to the control and # *p* < 0.05, ## *p* < 0.01, and ### *p* < 0.001, compared to the IFN-γ-treated group.

To investigate whether NLRP3 inflammasome activation was related to MSCT in hORSCs, we examined the expression of *NLRP3, Apoptosis-associated speck-like protein containing a CARD (ASC), caspase-1, and IL-1β* at the mRNA levels. As shown in Figure 2, *NLRP3, ASC, caspase-1, and IL-1β* were significantly increased in the IFN-γ-treated group compared to the controls. There was no significant difference in the expression levels in the MSCT group compared to the controls, whereas the expression of inflammasome components was upregulated in the IFN-γ-treated group, and downregulated by additional MSCT.

### 2.3. Effect of hHMSC Treatment on HF-IP Collapse-Related Genes

**Figure 3:**
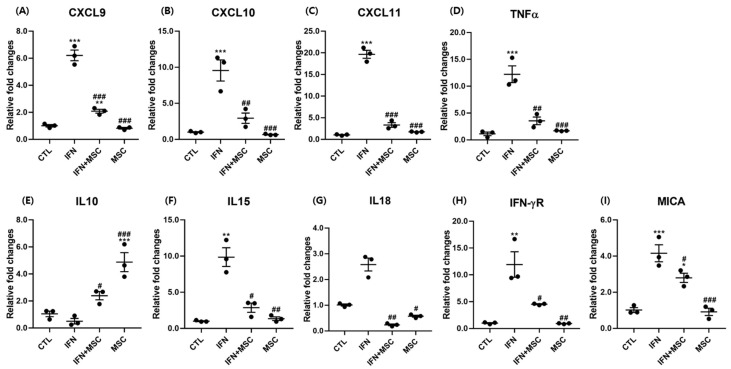
Analysis of gene expression by RT-PCR. The mRNA expression of (**A**) CXCL9, (**B**) CXCL10, (**C**) CXCL11, (**D**)TNF-α, (**E**) IL-10, (**F**) IL-15, (**G**) IL-18, (**H**) IFN-γR, and (**I**) MICA in hORSCs. HF-IP collapse-related genes were upregulated by IFN-γ and reverted by MSCT. Error bars represent the mean ± SEM, n = 3. Statistically significant at * *p* < 0.05, ** *p* < 0.01, and *** *p* < 0.001 compared to the control and # *p* < 0.05, ## *p* < 0.01, and ### *p* < 0.001 compared to the IFN-γ-treated group.

To further investigate the association between anagen arrest and re-entry by MSCT in hORSCs, we examined the expression *of CXCL9, CXCL10, CXCL11, TNF-α, IL-10, IL-15, IL-18, IFN-γR,* and Major histocompatibility complex (MHC) class I chain-related protein A (MICA) at the mRNA level (Figure 3). As shown in Figure 3, the hair follicle immune privilege (HF-IP) collapse-related gene (Figure 3A–I) expression levels were significantly increased in the IFN-γ-treated group compared to the controls. The changes in expression were not significantly different following MSCT compared to the controls. Compared to the IFN-γ-treated group, the expressions were suppressed by MSCT in the IFN-γ-hHMSC-treated group and the MSCT group. The expression level of the anti-inflammatory cytokine *IL-10* was upregulated by MSCT compared to the controls (Figure 3E). Changes in the expression of *IL-10* were not significant in the IFN-γ-treated group compared to the controls. Compared to the IFN-γ-treated group, *IL-10* expression was increased by MSCT in the IFN-γ-hHMSCs-treated group and the MSCT group.

### 2.4. Effect of hHMSC Treatment on Wnt/β-Catenin Signaling Pathway Molecules

**Figure 4:**
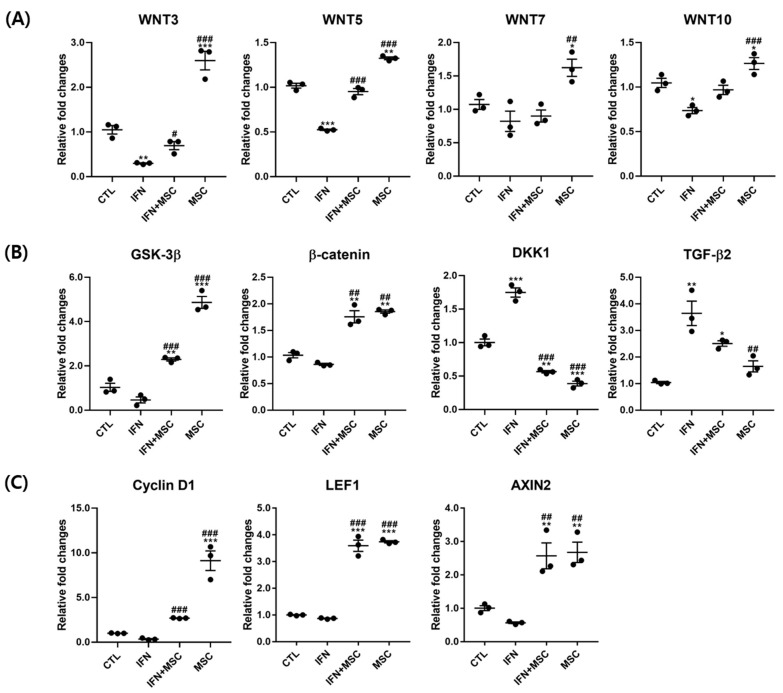
Analysis of gene expression by RT-PCR. The mRNA expression of (**A**) WNT3, WNT5, WNT7, WNT10, (**B**) GSK-3β, β-catenin, DKK1, TGF-β*2* and (**C**) Cyclin D1, LEF1, AXIN2 in hORSCs. Wnt/β-catenin-related genes were downregulated by IFN-γ and reverted by MSCT. DKK1, TGF-β*2* were inversely expressed. Error bars represent the mean ± SEM, n = 3. Statistically significant at * *p* < 0.05, ** *p* < 0.01, and *** *p* < 0.001 compared to the control and # *p* < 0.05, ## *p* < 0.01, and ### *p* < 0.001 compared to the IFN-γ-treated group.

We measured the expression of Wnt/β-catenin genes by real-time PCR to investigate the roles of MSCT (Figure 4). The results showed that MSCT significantly upregulated the mRNA expression levels of *WNT3, WNT5, WNT7, WNT10, GSK-3β, β-catenin*, *Cyclin D1, Lymphoid-enhancer factor-1 (LEF1)* and *AXIN2* compared to the controls. Additionally, IFN-γ-hHMSC-treated group increased the mRNA expression levels of *WNT3, WNT5, WNT10, GSK-3β, β-catenin, Cyclin D1, LEF1* and *AXIN2*, which was decreased by IFN-γ. The mRNA levels of *WNT7* were significantly increased only in the MSCT group.

As shown in Figure 4B, MSCT downregulated the expression levels of *DKK1* in the MSCT group with and without IFN-γ treatment compared to the controls. The change in *TGF-β2* expression was not significant in the MSCT group compared to the controls. The expression levels of *DKK1* and *TGF-β2* were increased in the IFN-γ-treated group and were suppressed by MSCT.

**Figure 5:**
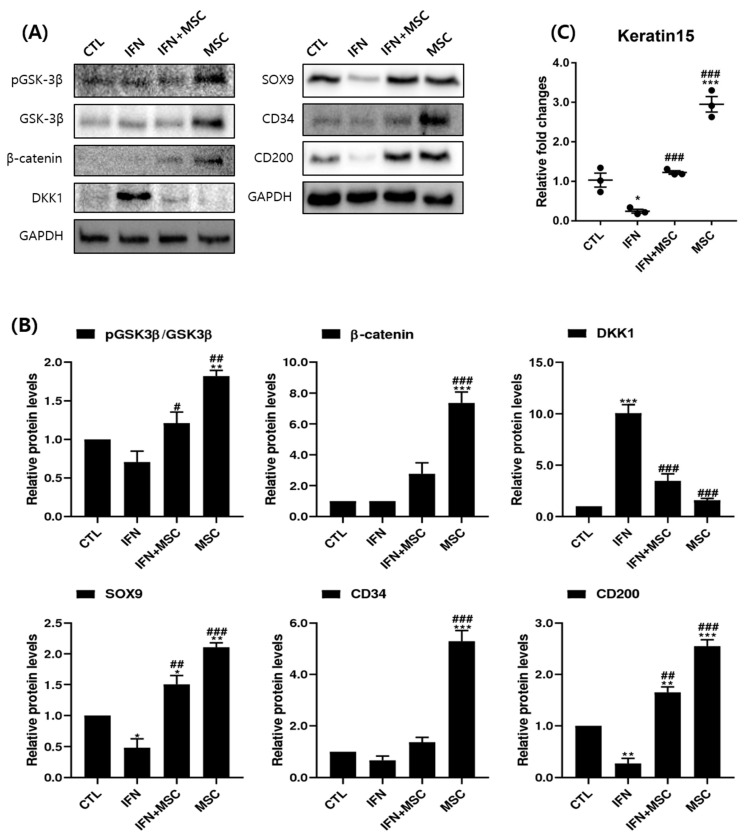
Analysis of protein levels of WNT/β-catenin pathway-related markers and hair stem cell markers induced by IFN-γ and MSCT. (**A**) Western blotting band images and (**B**) graphs of the quantitation of the Western blotting bands. Expression levels of the markers were assessed by Western blotting and GAPDH was used as a loading control. (**C**) Analysis of *Keratin 15* expression by RT-PCR. hHMSC treatment increased the protein expression of β-catenin, phosphorylated GSK-3β, SOX9, CD34, and CD200 compared to the controls and reverted the effect of IFN-γ in hORSCs. The levels of DKK1 were increased in the IFN-γ-treated group and reverted by MSCT. The levels of *Keratin 15* were downregulated by IFN-γ and reverted by MSCT. Error bars represent the mean ± SEM, n = 3. Statistically significant at * *p* < 0.05, ** *p* < 0.01, and *** *p* < 0.001 compared to the control and # *p* < 0.05, ## *p* < 0.01 and ### *p* < 0.001 compared to the IFN-γ-treated group.

We also investigated the effects of MSCT on the WNT/β-catenin pathway at the protein level by Western blotting (Figure 5). The levels of β-catenin and phosphorylation of GSK-3β were increased in the MSCT group compared to the controls. Compared to the IFN-γ-treated group, β-catenin and the phosphorylation of GSK-3β were upregulated by MSCT in the IFN-γ-hHMSC-treated group and the MSCT group. In contrast, the level of DKK1 was increased in the IFN-γ-treated group compared to the controls, and DKK1 was significantly reverted by MSCT. The protein expressions were similar to the mRNA levels (Figure 4).

### 2.5. Effect of hHMSC Treatment on Hair Stem Cell Markers

We investigated the effects of MSCT on hair stem cell markers at the protein expression level (Figure 5). Whereas the levels of SRY-Box Transcription Factor 9(SOX9), CD34, and CD200 were significantly decreased in the IFN-γ-treated group compared to the controls, their levels were significantly increased in the MSCT group compared to the controls. The expression of SOX9 and CD200 was upregulated by MSCT in the IFN-γ-hHMSC-treated group and the differences were statistically significant compared to the IFN-γ-treated controls (Figure 5B). CD34 protein expression was increased by additional MSCT in the IFN-γ-treated group, but the change was not significant (Figure 5). We also measured the expression of *Keratin15* genes by qPCR (Figure 5C). The results showed that MSCT significantly upregulated the mRNA expression levels of *Keratin15* compared to the controls. Additionally, IFN-γ-hHMSC-treated group increased the expression level of mRNA of *Keratin15*, which was decreased by IFN-γ.

### 2.6. Effect of hHMSC Treatment on Growth Factors

Next, we examined the growth factors at the mRNA expression level. Insulin-like growth factor 1(IGF1), fibroblast growth factor 2(FGF2), fibroblast growth factor 7(FGF7), vascular endothelial growth factor (VEGF), platelet-derived growth factor (PDGF), sonic hedgehog (SHH), bone morphogenetic protein 2 (BMP2), and BMP4 are representative growth factors associated with hair growth.

**Figure 6:**
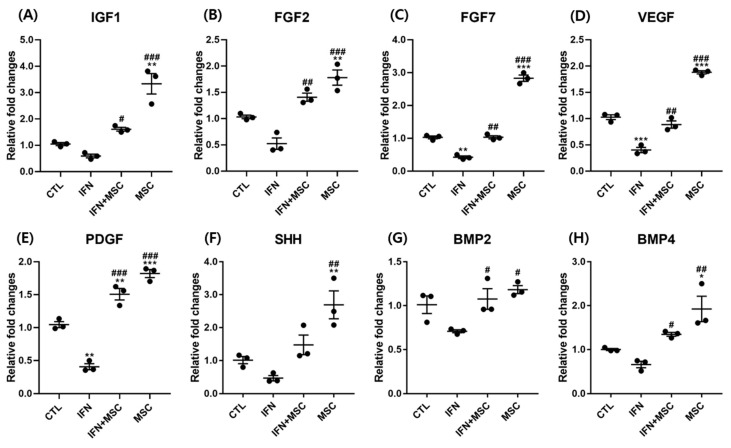
Analysis of gene expression by RT-PCR. Effects of hHMSC treatment on hair-growth-related growth factors. hHMSC treatment significantly upregulated the mRNA of various growth factors including (**A**) IGF1, (**B**) FGF2, (**C**) FGF7, (**D**) VEGF, (**E**) PDGF, (**F**) SHH, (**G**) BMP2 and (**H**) BMP4 which were suppressed by IFN-γ. Error bars represent the mean ± SEM, n = 3. Statistically significant at * *p* <0.05, ** *p* < 0.01 and *** *p* < 0.001, compared to the controls and # *p* < 0.05, ## *p* < 0.01 and ### *p* < 0.001 compared to the IFN-γ-treated group.

As shown in Figure 6, the expression levels of FGF7, VEGF, and PDGF were significantly suppressed in the IFN-γ-treated group compared to the controls. The expression levels of IGF1, FGF2, SHH, BMP2 and BMP4 were suppressed by IFN-γ treatment but the change was not significant compared to the controls. Interestingly, the levels of all growth factors including *IGF1, FGF2, FGF7, VEGF, PDGF, SHH, BMP2* and *BMP4* in response to MSCT were significantly upregulated compared to the control or IFN-γ-treated groups in this study.

### 2.7. Effect of hHMSC Treatment on the JAK/STAT Pathway

**Figure 7:**
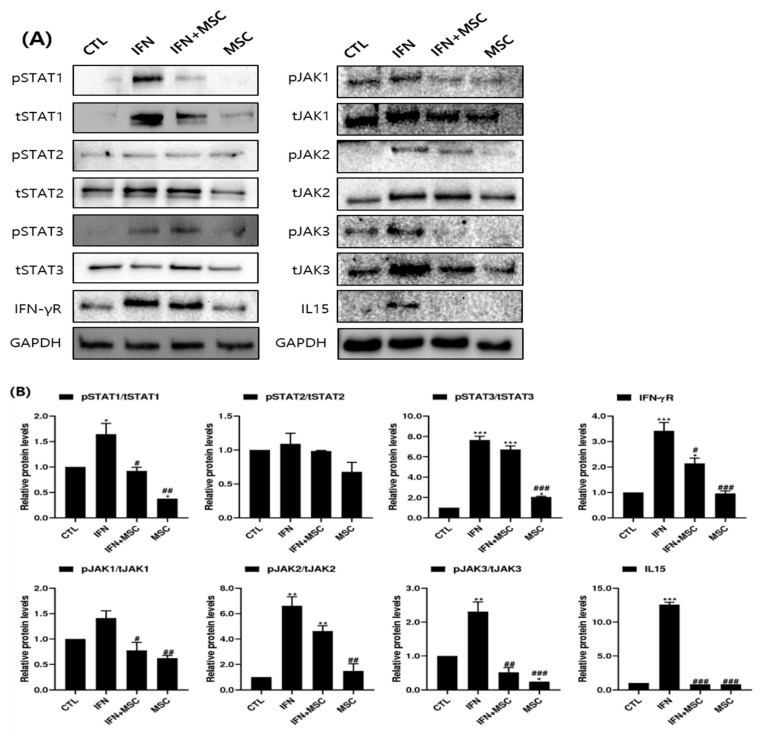
Analysis of JAK/STAT pathway-related marker protein levels induced by IFN-γ and MSCT. (**A**) Western blotting band images and (**B**) graphs of Western blotting band quantitation. The expression levels of JAK/STAT pathway molecules were assessed by Western blotting. GAPDH was used as a loading control. Overall, the expression of proteins in the IFN-γ-treated group was upregulated compared to the controls and downregulated by MSCT. Error bars represent the mean ± SEM, n = 3. Statistically significant at * *p* < 0.05, ** *p* < 0.01, and *** *p* < 0.001 compared to the control and # *p* < 0.05, ## *p* < 0.01, and ### *p* < 0.001 compared to the IFN-γ-treated group.

We examined the effects of MSCT on the JAK/STAT signaling pathway at the protein level using Western blotting (Figure 7). Overall, the expression of JAK1, JAK2, JAK3 and STAT1, STAT2, STAT3 proteins in the IFN-γ-treated group was upregulated compared to the controls, and phosphorylation and some total protein levels in the Western blots were downregulated by MSCT (Figure 7A).

The level of phosphorylation of JAK1-3 was significantly increased in IFN-γ-treated cells compared to the controls. Phosphorylation was significantly suppressed by MSCT compared to the IFN-γ-treated controls (Figure 7B). The levels of phosphorylated STAT1 and STAT3 were also increased by IFN-γ treatment, and the expression of STAT1 phosphorylation was decreased by additional MSCT. Whereas the expression of STAT3 was suppressed by MSCT compared to controls, the change in IFN-γ-hHMSC-treated group was not significant compared to both controls and IFN-γ-treated group. The effect of IFN-γ or MSCT on the expression of STAT2 was not remarkable.

Next, we also investigated the effect of MSCT on IFN-γR and IL-15, which are the key cytokines in JAK/ STAT pathway activation. The level of IFN-γR was increased in the IFN-γ-treated group compared to the control and was suppressed by MSCT. IL-15 was only expressed in the IFN-γ-treated group and MSCT blocked the expression of IL-15 (Figure 7).

### 2.8. Effect of hHMSC Treatment on hORSC Migration Ability

**Figure 8:**
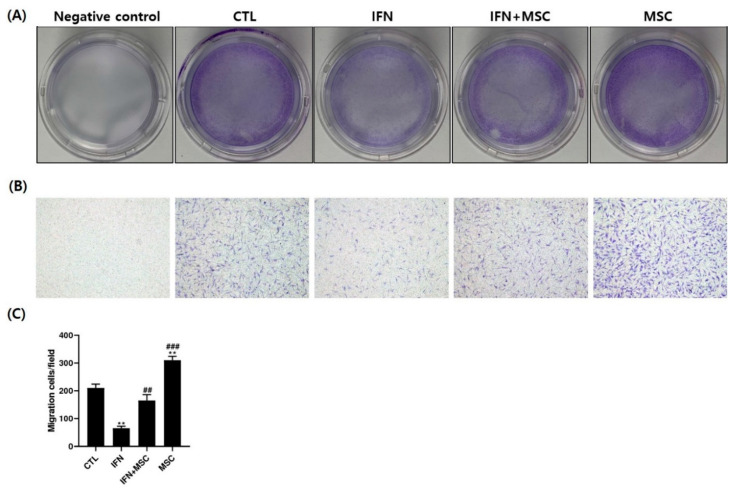
Transwell migration assay. The effect of hHMSC co-culture on hORSC migration was examined using the crystal violet assay. (**A**) Macroscopic observation of the Transwell chamber containing the negative control. (**B**) Images of migrated cells stained by crystal violet at ×100 magnification. (**C**) Quantification of the migrated cells. Cell migration ability was decreased in the IFN-γ-treated group but increased in the hHMSC co-culture group compared to the control. Compared to the IFN-γ-treated group, the migration ability of the IFN-γ-hHMSC-treated group and the hHMSC co-culture group was significantly increased. Error bars represent the mean ± SEM, n = 3. Statistically significant at ** *p* < 0.01 compared to the control and ## *p* < 0.01 and ### *p* < 0.001 compared to the IFN-γ-treated group.

Next, the effect of MSCT on hORSC migration was examined by the crystal violet assay using six-well polycarbonate Transwell inserts. As shown in Figure 8, cell migration ability was decreased in the IFN-γ-treated group but increased in the hHMSC co-culture group compared to the controls. Compared to the IFN-γ-treated group, the migration ability of the IFN-γ-hHMSC-treated group and the hHMSC-only group was significantly increased. The quantitative determination of transferred cells is shown in Figure 8C.

### 2.9. Effects of hHMSCs Treatment on the Mouse Vibrissa HF Organ Culture

To examine the effect of MSCT at the organ level, we performed an ex vivo culture of mouse vibrissae HFs. In this study, 20 HFs were cultured individually with control, IFN-γ, IFN-γ plus MSCT, or MSCT. Each hair shaft was measured every third day.

**Figure 9:**
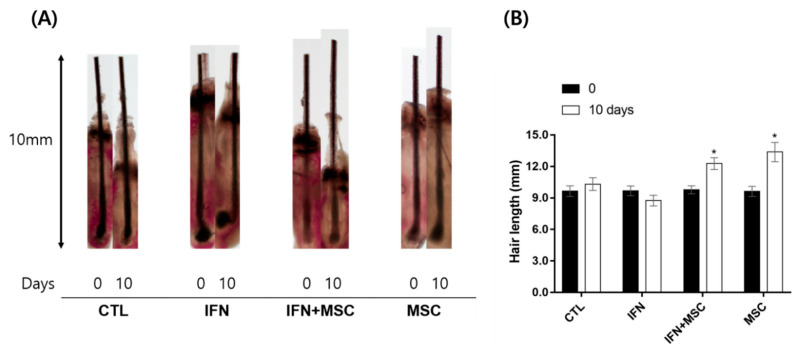
Mouse vibrissae organ culture. (**A**) HF and hair shaft morphology during the 10-day experimental period. (**B**) Hair length of hair shafts of 0 and 10 days. MSCT stimulates hair shaft and HFs in organ culture. A total of 80 mouse vibrissae were obtained and treated for 10 days with IFN-γ (100 ng/mL), MSCT, or both IFN-γ and MSCT. The length of each HF was measured under a microscope on days 0 and 10. The relative length of each hair shaft is shown as mean ± SEM (upper panel). Error bars represent the mean ± SEM, n = 5. * *p* < 0.05 compared with the 0 day.

As shown in Figure 9, HFs and the hair shaft in the MSCT group grew longer than the control, and hair bulb structure was preserved at 10 days. The hair shaft of the IFN-γ-treated group did not grow longer than control and the hair bulb was changed. We observed the hair separated from the follicle and the morphology of the hair bulb was curved, which are catagen-like regressive changes. The IFN-γ-hHMSC-treated group also showed growth of the hair shaft compared to the control. Compared to the IFN-γ treated group, the hair bulb of the IFN-γ-hHMSC-treated group was preserved and the hair shaft was longer.

## 3. Discussion

hHMSCs have received attention as a promising therapeutic strategy because of their immunomodulatory effects on various types of cells [20,21,22,23,24]. We wanted to investigate how MSCT could affect anagen re-entry signaling and hair growth-promotion in IFN-γ-treated hORSCs, which is similar to the AA microenvironment. The results showed that MSCT upregulated hORSC viability, migration, and the expression of anagen-re-entry-related genes, unlike IFN-γ treatment (Figure 1, Figure 3 and Figure 8). We found that the NLRP3 inflammasome components highly expressed after IFN-γ treatment were efficiently suppressed by MSCT (Figure 2). IFN-γ signature genes and JAK/STAT-related pro-inflammatory cytokines induced by IFN-γ were decreased by MSCT. The anti-inflammatory cytokine *IL-10*, which contributes to the immunomodulatory effects of MSCT, was increased by MSCT. Hair growth factors were upregulated by MSCT (Figure 6). The immunomodulatory effects of MSCT on hORSCs appeared to be associated with Wnt/β-catenin stimulation and inhibition of the JAK/STAT signaling pathway (Figure 4, Figure 5 and Figure 7).

Inflammatory cytokines, such as IFN-γ, are known to lead to the collapse of HF-IP by upregulation of expression of MHC class I a and MICA inside the HF.

MICA is a polymorphic protein that is induced upon stress, damage or transformation of cells. In AA, NKG2D+ Natural killer cells (NK cells) attack MICA positive HFs, which results in apoptosis and hair loss [25]. According to studies, upregulation of MICA in lesional AA enhances the susceptibility of attack by NK cells on the HFs, which may then promote anagen termination and AA progression. MSCs have a suppressive effect on the proliferation of activated helper T cells, caused by cell cycle arrest in the G0/G1 phase [26]. The inhibition of T cell proliferation reduces IFN-γ secretion by Th1 cells and IL-17 secretion from Th17 cells and increases IL-4, IL-10 secretion by Th2 cells. In this study, MICA was significantly induced by IFN-γ in hORSCs but downregulated by MSCT (Figure 3). This may suggest that MSCT inhibits the expression of IFN-γ induced MICA in hORSCs.

The Wnt/β-catenin signaling pathway is important for hair morphogenesis and anagen re-entry [27]. β-catenin is required to increase the proliferation of bulge stem cells [28]. Cyclin D1 is a member of the cyclin protein family that is involved in regulating cell cycle progression. It is known to increase during the G0/G1 phase and to initiate DNA synthesis. Cyclin D1 and Axin2 are one of the target genes of the Wnt/β-catenin signal pathway. They are direct downstream molecules activated by β-catenin. LEF1 is a transcription factor that is primarily involved in the canonical Wnt/β-catenin signaling pathway [29]. They are also important for anagen re-entry and cell proliferation.

In this study, MSCT significantly induced Wnt family proteins, β-catenin, GSK-3β and cyclin D1 expression. Among the Wnt family proteins, Wnt3a and 10b are related to the hair cycle and regeneration, and increase anagen gene expression. Kishimoto et al. generated Wnt3a-overexpressing keratinocyte feeder cells and co-cultured the cells with freshly isolated murine DPCs. In this experiment, Wnt3a was found to increase β-catenin expression and increase hair growth in nude mice that underwent skin remodeling composed of DPCs and keratinocytes [30]. Wnt5a is known as a downstream molecule of the sonic hedgehog signaling cascade and is upregulated during the early morphogenetic stages [31,32]. We also demonstrated that MSCT suppressed the expression of DKK1 in IFN-γ-induced hORSCs. However, this did not seem to be caused by TGF-β2. The reason for this is that *TGF-β2* mRNA was only increased by IFN-γ treatment and not by MSCT in this study. Most of the Wnt/β-catenin-related molecules were increased by MSCT conditions in vitro. This suggests that MSCT could help induce anagen re-entry by stimulating the Wnt/β-catenin signal pathway.

SOX9 is characteristically expressed in CD34-positive hORSCs in the bulge area and is involved in the regulation of bulge formation and stem cell transcription [33,34,35]. CD34 and CD200 are known as human follicular stem cell markers. Keratin15 is one of the most widely used markers of bulge stem cells [36]. Since SOX9 is known as a β-catenin-regulated gene, the MSCT-induced enhanced expression of SOX9 may result from the activation of Wnt/β-catenin signaling by MSCT. CD200 suppresses the secretion of the pro-inflammatory cytokine TNF-α and improves the secretion of the anti-inflammatory cytokine IL-10. The expression of SOX9, CD34, CD200, and Keratin15 protein was increased after hHMSC co-culture (Figure 5). This supports that MSCT could strengthen the stemness of HF stem cells in the bulge area of hORSCs [30].

In AA, dystrophic HFs are often observed instead of normal HFs because hORSCs are attacked by inflammatory cells around the HFs and the immune privilege of the HFs is disrupted. The main role of hORSCs is to secrete and react to inflammatory substances. hORSCs are important immunocompetent cells that express NLRP3 inflammasomes constitutively in the HFs [19]. Therefore, we investigated the MSCT response with inflammasome markers, which are closely related to hORSCs. In our study, the inflammasome components were upregulated by IFN-γ treatment and downregulated by MSCT (Figure 2). This finding may suggest new ways for regulating the inflammasome activation pathway and represent a way to prevent or treat AA.

We also found that the IFN-γ-induced proinflammatory cytokines and chemokines of AA (Figure 3) were suppressed by MSCT in hORSCs [37,38]. Due to the inflammatory response, these molecules disrupt the immune privilege of HFs, induce dystrophic change and catagen, and prevent anagen re-entry. In contrast, *IL-10* is an anti-inflammatory cytokine that has the opposite effect. These results can be the outcome of JAK/STAT pathway activation. Recent studies have shown that increased JAK-STAT signaling suppressed HF stem cell function in vitro and that STAT5 signaling regulated HF stem cell quiescence [39,40]. Even if the JAK/STAT signaling pathway is important in skin inflammation, the role of JAK/STAT signaling in hORSCs has not yet been studied.

In this study, the phosphorylation of JAK1-3 and STAT1 was significantly decreased in hORSCs by MSCT, which reverted the IFN-γ-induced proinflammatory changes. Although the downregulation of STAT3 in the IFN-γ-hHMSCs treated group was not significant compared to the IFN-γ-treated groups, the changes were significant compared to the controls in Western blotting analysis. We suggest that MSCT suppress IFN-γ signaling through downregulation of the JAK/STAT pathway. This result suggests that the MSCT-suppressed activation of JAK1/2 reversed catagen induction and induced a potential mechanism involved in the new hair cycle. TGF-β is well known as an inducer of regression [41]. In accordance with that, the TGF-β2 mRNA in hORSCs was significantly upregulated in response to IFN-γ treatment and was reduced by MSCT in this study.

We also found a significant increase in the mRNA expression of hair growth factors in hORSCs by MSCT. IGF-1 is a well-known anagen-prolonging factor and restored the immune activity of hair follicles [42]. PDGF is related to the induction and maintenance of the anagen phase, and FGF and is known as a hORSC growth factor. VEGF is involved in accelerating hair growth, increasing follicle size through follicular angiogenesis and stimulating ORSCs migration [43,44]. SHH has been found to regulate the angiogenic growth factor such as VEGF. BMP2, BMP4 activation is known to elaborately regulate the development of HF bulge stem cell activation and growth during the hair cycle [45]. hHMSCs secrete several growth factors such as VEGF, and FGF, which lead to escalated cell proliferation, regeneration and promote the migration of cells. As shown in Figure 6, *IGF1, FGF2, FGF7, PDGF*, *VEGF, SHH, BMP2* and *BMP4* mRNA expression was suppressed by IFN-γ, and MSCT had a stimulatory effect on the transcription of growth factors. Our results indicate that MSCT increased hair growth factors to facilitate the viability and migration of hORSCs (Figure 1 and Figure 8).

We previously studied the efficacy of MSCT in hDPCs, one of the key cells that make up the hair bulge [37]. There are similarities and differences between the results of that study and the results of the current research. As for the similarities, in hDPCs, MSCT also reduced the inflammatory markers *caspases-1* and *IFN-γR*. In addition, the Wnt/β-catenin signaling pathway markers Wnt10, β-catenin, and pGSK-3β increased, and DKK1 decreased in both cells. The growth factor *FGF2* increased and the catagen inducer *TGF-β2* decreased, suggesting that in hDPCs and hORSCs, hHMSCs treat inflammation-related substances and are involved in the regulation of Wnt/β-catenin signaling and promote the secretion of growth factors. As for the differences, the inflammatory substances *IL-15, TNF-α,* and *IL-1β* were increased in hDPCs after hHMSCs treatment, whereas there was a clear decrease in cytokines in the hORSCs. Since cells secreting inflammatory substances such as IL-15 and TNF-α are mainly hORSCs, it can be inferred that MSCT could be relatively safe to use for HFs in vivo based on this result. Additionally, there was a clear difference in the JAK/STAT pathway-related results. In hDPCs, MSCT increased the expression levels of STAT1 and STAT3 compared to the IFN-γ-treated group. Thus, the JAK/STAT pathway acted as an anagen re-entry signal here. In contrast, in this study, MSCT in hORSCs reduced the expression levels of STAT1 and STAT3, which subsequently offset the proinflammatory effect of IFN-γ. This result may be because MSCT in hORSCs acted as a pro-inflammatory effect on the JAK/STAT pathway. The different interactions seemed to result from the different properties of each cell type.

In our study, the migration ability of hORSCs was increased by MSCT, which was decreased by IFN-γ treatment (Figure 8). The migration ability of hORSCs is one of the important factors in HFs growth. hORSCs migration is necessary and sufficient to proceed with the hair cycle. hORSCs represent a very proliferative compartment in the HFs during the anagen phase [38]. This is an important part of hair growth in the hair cycle.

As shown in Figure 6, MSCT increases the expression level of hair growth factors related with cell migration in hORSCs. In addition, hHMSCs secretes multiple growth factors, which help cell proliferation and migration. Therefore, this result means that MSCT can play an active role in HFs growth by enhancing cell viability and migration under the microenvironment with HF-IP collapse.

In this study, we demonstrated that MSCT increase hair shaft growth in mouse vibrissae organ culture (Figure 9). As shown in Figure 9, hair bulbs were preserved in the MSCT treated group contrary to the IFN-γ group, in which the hair bulb was curved. This is one of the catagen-like regressive changes in hair bulb. Our results suggest that MSCT increases hair growth factors (Figure 6) and stimulates Wnt/β-catenin signaling to stimulate cyclin D1 (Figure 4) and suppress the catagen inducer TGF-β2. MSCT also blocks the proinflammation effects of IFN-γ through inhibition of the JAK/STAT pathway. Through these pathways, anagen re-entry and anagen prolongation would be improved in HF.

Recent studies have shown that induced pluripotent stem cells can differentiate into MSCs to have DPC characteristics when exposed to a certain environment [46]. These MSCs were able to partially reproduce the characteristics of DPC, such as showing a DPC-related marker and raising HF-related genes. In addition, amniotic fluid-derived mesenchymal stem cell-conditioned medium promoted telogen–anagen conversion in HFs and increased HF density, mimicking the role of DPC [47]. Originally, MSCs and DPCs are from the mesoderm, which are different from hair keratinocytes which are ectodermal in origin [48]. Based on these studies, we predict that MSCs could change to have the ability to activate the Wnt/β-catenin signaling pathway and to induce anagen-reentry by modifying self-characteristics, similarly to DPCs in certain aspects, when co-cultured with hair keratinocytes.

In association with AA, the effect of MSCT on hORSCs was first investigated in this study. Our results show that MSCT could induce anagen re-entry-related molecules in hORSCs. MSCT may induce anagen re-entry through activation of Wnt/β-catenin, inhibition of the JAK/ STAT pathway, HF-IP collapse-related genes, and NLRP3 inflammasome activation. Various growth factors related to AA were enhanced in response to MSCT. MSCT could increase hORSC viability and migration which results in hair shaft growth. MSCT can be an attractive treatment for refractory AA patients at various stages. Additional research is needed to determine the optimal clinical setting for MSCT.

## 4. Materials and Methods

### 4.1. Cell Culture

We obtained human bone marrow-derived mesenchymal stem cells (Catholic MASTER Cells from the Catholic Institute of Cell Therapy (CIC, Seoul, Korea). We purchased human outer root sheath cells (hORSCs) from ScienCell (San Diego, CA, USA) and cultured them, as previously described [49]. hORSCs (1 × 10^5^ cells per well) were seeded into six-well culture plates in Dulbecco’s Modified Eagle Medium (DMEM high glucose, Gibco BRL, Life Technology, Karlsruhe, Germany) containing 10% fetal bovine serum (FBS; Gibco BRL, Life Technology, Karlsruhe, Germany) and 1% penicillin/streptomycin (Gibco, BRL) for 24 h. hORSCs at passage three to five were used for the experiments. Then, hORSCs were seeded on culture dish plates with serum-free DMEM, and the plates were co-cultured with upper chamber containing hHMSCs.

### 4.2. Co-Culture

The co-culture of hORSCs and hHMSCs was performed as previously described [50]. hORSCs were seeded in the lower chambers of Transwell culture plates (Cell Culture Insert Companion Plate; Corning, Falcon, Franklin Lakes, NJ, USA) at a density of 1 × 10^5^ cells per well. hHMSCs were added to the upper chamber of the Transwell in separate plates at a density of 5 × 10^4^ cells per well. The upper chambers were coated with polyethylene terephthalate (pore size: 0.8 mm, six-well format, Cell Culture Inserts; Corning, Falcon). After 24 h, the media was changed to serum-free DMEM and the upper chamber containing hHMSCs was transferred to the wells where the hORSCs were cultured to produce the co-culture. The IFN-γ group was treated with recombinant human IFN-gamma at 100 ng/mL, obtained from Peprotech (315-05, Rocky Hill, NJ, USA). After different times (24 h, 48 h, and 72 h), the upper chamber was removed and washed with Dulbecco’s phosphate-buffered saline (DPBS, T&I, Bioprince, Chuncheon, Korea). Next, the hORSCs were harvested and used for analysis. The experiment was repeated three times.

### 4.3. Cell Viability Assay (MTT Assay)

Cell viability was measured via the 3-(4,5-dimethylthiazol-2-yl)-2,5-diphenyltetrazolium bromide (MTT) assay. After co-culturing for different times, the MTT reagent was added and reacted for 2 h. The reagent was removed, and the resulting formazan crystals were dissolved in dimethylsulfoxide (DMSO) [51]. Then, the samples were assessed by measuring the absorbance at 540 nm with an enzyme-linked immunosorbent assay (ELISA) plate reader.

### 4.4. Real-Time PCR

Total RNA was isolated from the hORSCs using TRIzol reagent (Invitrogen, Carlsbad, CA, USA), and cDNA was synthesized with the MG cDNA Synthesis Kit (CancerROP, Seoul, Korea) according to the manufacturer’s instructions. Total RNA (1 μg) was reverse transcribed with primers and Moloney-murine leukemia virus (M-MLV) reverse transcriptase (RTase) (CancerROP, Seoul, Korea). Real-time PCR was performed using the SYBR Green Master mix (CancerROP, Seoul, Korea). The gene expression levels were quantified using analysis software (Quantity One 1-D analysis, Bio-Rad, Hercules, CA, USA). The primer sequences are listed in Supplementary Table S1. The primers were designed and obtained from Bioneer (Bioneer, Daejeon, Korea).

### 4.5. Western Blot Analysis

hORSCs were harvested with RIPA lysis buffer supplemented with Protease and Phosphatase Inhibitor Cocktail (ThermoFisher, Rockford, IL, USA). The amount of protein was measured using the BCA protein assay kit (ThermoFisher, Rockford, IL, USA) and compared to bovine serum albumin (ThermoFisher) standards. Cell lysates containing the same quantity of total protein were separated by electrophoresis on sodium dodecyl sulfate (SDS)-polyacrylamide gels and transferred to polyvinylidene fluoride (PVDF) membranes. The membranes were blocked with 5% skim milk in TBST for an hour at room temperature and incubated with primary antibodies in 5% BSA at 4 °C overnight. Primary antibodies against phospho-JAK1-3, total-JAK1-3, phospho-STAT1-3 total-STAT1-3, DKK1, IL-15, phospho-GSK-3β, GSK-3β, β-catenin, SOX9, CD34, CD200, and GAPDH were purchased from Santa Cruz Biotechnology (Santa Cruz, CA, USA) and Cell Signaling Technology, Inc. (Cell Signal, Beverly, MA, USA). On the following day, the membranes were washed with TBST several times and incubated in peroxidase-conjugated secondary antibody (Cell Signaling Technology) for 2 h at room temperature. The immunoreactive bands were visualized by the enhanced chemiluminescence (ECL) detection system (ThermoFisher) and were obtained using a chemiluminescence imaging system (ChemiDoc Imaging System; Biorad, Hercules, CA, USA) after incubation with horseradish peroxidase.

### 4.6. Transwell Migration Assay

The migration of hORSCs was measured using Transwell plates that were 6.5 mm in diameter with 8 μm pore filters. The Transwell system was separated from the lower segment by a permeable membrane coated with polyethylene terephthalate. The upper chambers were loaded with 1 × 10^5^ hORSCs that were already treated with IFN-γ or hHMSCs. The number of hHMSCs and density of IFN-γ were the same as those used in the co-culture procedure. DMEM containing 10% FBS was added to the lower chambers. After incubation for 15 h at room temperature, the cells in the upper chamber were carefully removed using a cotton swab and the membranes were fixed with 4% paraformaldehyde for 20 min. The cells that had migrated under the filter were stained with 0.5% crystal violet for 20 min and then observed under a light microscope [52]. The cells in five randomly selected visual fields under each membrane were counted.

### 4.7. Hair Follicle Organ Culture Assay

Six-week-old female C57BL/6 mouse vibrissae follicles were micro-dissected under sterile conditions, as previously described [9]. Individual follicles were placed in a 24-well tissue culture plate in Dulbecco Modified Eagle Medium (DMEM high glucose, Gibco BRL, Life Technology, Karlsruhe, Germany), which was supplemented with hydrocortisone, insulin, and glutamine in the presence of IFN-γ (100 ng/mL) or hHMSCs. hHMSCs were added to the Transwell upper chamber in separate plates at a density of 1 × 10^4^ cells per well as described in the co-culture method. At least 20 HFs were cultured individually with control, IFN-γ, IFN-γ plus MSCT, or MSCT. The culture medium was changed every three days, and images of individual follicles were filmed before and after hHMSCs treatment. All procedures were carried out under the Institutional Animal Care and Use Committee—approved protocols (CUMC-2019-0061-02, date of approval: 24 July 2019).

The length of vibrissae HFs from the top of hair to the bottom of the hair bulb in organ culture was measured with a microscope every third day for 10 days. Changes in hair length were calculated from the photographs and expressed as mean ± SEM of 20 vibrissae HFs.

### 4.8. Statistical Analysis

All data are expressed as the mean ± SEM. An ANOVA test and paired Student’s *t*-test were used for the statistical analyses with SPSS (v.7.0) software (IBM Corp., Armonk, NY, USA). All tests were one-sided, and a *p*-value of less than 0.05 was considered statistically significant.

## Data Availability

The data that support the findings of this study are available from the corresponding author upon reasonable request.

## Supplementary Materials

The supplementary materials are available online at https://www.mdpi.com/article/10.3390/ijms22094581/s1.

## Abbreviations

AA	Alopecia areata
hORSCs	Human outer root sheath cells
hHMSCs	Human hematopoietic mesenchymal stem cells
hDPCs	Human derma papilla cells
MSCT	Mesenchymal stem cell therapy
JAK	Janus kinase
STAT	Signal transducers and activators of transcription protein
CXCL	C-X-C motif chemokines
IL	Interleukin
IFN	Interferon
FBS	Fetal bovine serum
DMEM	Dulbecco Modified Eagle Medium
MTT	3-(4,5-dimethylthiazol-2-yl)-2,5-diphenyltetrazolium bromide
RT-PCR	Real time-polymerase chain reaction
HFs	Hair follicles
HF-IP	Hair follicle-immune privilege
NLRP3	NLR family pyrin domain containing 3
ASC	Apoptosis-associated speck-like protein containing a CARD
MICA	Major histocompatibility complex (MHC) class I chain-related protein A
NK cell	Natural killer cell
LEF1	Lymphoid enhancer binding factor 1
AXIN2	Axis inhibition protein 2
BMP2	Bone morphogenetic protein
SHH	Sonic hedgehog
ALP	Alkaline phosphatase
CRABP1	Cellular retinoic acid binding protein 1
SOX	SRY-box transcription factor
DP	Dermal papilla
