# Supplementary material for: Mesenchymal Stem Cells Antagonize IFN-Induced Proinflammatory Changes and Growth Inhibition Effects via Wnt/β-Catenin and JAK/STAT Pathway in Human Outer Root Sheath Cells and Hair Follicles
Source: Int J Mol Sci. 2021 Apr 27;22(9):4581. doi: 10.3390/ijms22094581 (PMC8123883; doi:10.3390/ijms22094581)
Supplement: Supplementary file 1 [file ijms-22-04581-s001.zip › ijms-1146545-supplementary.pdf]

Supplementary Materials

Mesenchymal Stem Cells antagonize IFN-induced proinflammatory changes and growth inhibition effects via Wnt/ β-catenin and JAK/STAT pathway in Human Outer Root Sheath Cells and Hair Follicles

All primers (**Table S1**) were designed using the coding sequences available on the GenBank database (http://www.ncbi.nlm.nih.gov/Genbank/Genbank Search.html) and were synthesized by Bioneer custom oligo synthesis service (Bioneer, Daejeon, Korea).

Table S1. Primer sequence

Species	Primer name	Forward (5'-3')	Reverse (5'-3')
Human	NLRP3	ACA AGC CAC CTC ACT TCC AG	CCA ACC ACA ATC TCC GAA TG
	ASC	TCA CAG AAG TGG ACG GAG TG	TGT CTT GGC TGG TGG TCT CT
	caspase-1	TAT CCG TTC CAT GGG TGA AG	TCA AAG CTC GGG TCT TAT CC
	IL-1β	GCC AAT CTT CAT TGC TCA AGT	ACT TCA TCT GTT TAG GGC CA
	CXCL9	GAC CTT AAA CAA TTT GCC CCA AG	TCC TTC ACA TCT GCT GAA TCT GG
	CXCL10	GCA AGC CAA TTT TGT CCA CG	ACA TTT CCT TGC TAA CTG CTT TCA G
	CXCL11	GAG CAG CAA AGC TGA AGT AG	AGG GCC TAT GCA AAG ACA G
	TNFα	CAC CAC TTC GAA ACC TGG GA	AGG AAG GCC TAA GGT CCA CT
	IL-10	GCT GAG AAC CAA GAC CCA GAC A	CGG CCT TGC TCT TGT TTT CA
	IL-15	GCC TTC ATG GTA TTG GGA A	TGC TGT TAC TTT GCA ACT GG
	IL-18	AGC TGA AGA TGA TGA AAA CCT G	ATA GAG GCC GAT TTC CTT GG
	IFN-γR	GCA TGG CTC TCC TCT TTC TC	CTG TGG CAT GAT CTG GTA CT
	MICA	GAA TCC GGC GTA GTC CTG AG	TCC GGG GAT AGA AGC TGG AA
	WNT3	ATG AAC AAG CAC AAC AAC GAG	TTG AGG AAG TCG CCG ATA G
	WNT5	CCC TGT ACA GAG ACC CGA GA	ACA ACT GGC ACA GCT TCC TC
	WNT7	TCT GTA ACA AGA TCC CAG GC	CAT TGC GGA ACT GAA ACT GA
	WNT10	CCC GGG ACA TCC AGG CGA GA	CTC TGG CGC TGC CCT CCA AC
	GSK-3β	CTT GGA CAA AGG TCT TCC GGC C	GTT GGC AGG CGG TGA AGC AG
	β-catenin	TTT AAG CCT CTC GGT CTG TG	CAA ATA CCC TCA GGG GAA CA
	Cyclin D1	GAC AGA CCG CGG CTC CTT	CAA CGT GAA TCT GGT TCC GA
	DKK1	TGT GCT AGA CAC TTC TGG TC	TTC TCC ACA GTA ACA ACG CT
	TGF-β2	AAC TGT CTG CCC AGT TGT TA	GCT GAG ACG TCA AAT CGA AC
	LEF1	CAG GTA CAG GTC CAA GAA TG	CTA GCA GTG ACC TCA GGG TA
	AXIN2	GCA GTA AGA AAC AGC TCC AG	CCC TCT CTC TCT TCA TCC TC
	KRT15	GGT GTA GGG TCT GAA GAA CA	CCA CAC CAG AAG AGT AAA GC
	IGF1	AAT CCC TCT TCT GCT TGC TA	ATT TTC CCC ATC GCT TCT GA
	FGF2	AGA AGA GCG ACC CTC ACA TC	ACT GCC CAG TTC GTT TCA GT
	FGF7	AAT TGT GGC AAT CAA AGG GG	CCG TTG TGT GTC CAT TTA GC
	PDGF	TGA TCT CCA AAC GCC TGC T	TCA TGT TCA GGT CCA ACT CG
	VEGF	TGC CCG CTG CTG TCT AAT	TCT CCG CTC TGA GCA AGG
	SHH	GAG ATG TCT GCT GCT AGT CC	GGA GAT CTT CCC TTC ATA CC
	BMP2	GCT GTA AGA GAC ACC CTT TG	GAG TTG ACC AAC GTC TGA AC
	BMP4	CAG GAG AGC AGA AAC AAG AG	CTC ACT CTC TCT GGA CCT CA
	APLP	TGT GGA GTA TGT GTG CTG TC	GTT TCC TCT TCC TCT TCA GC
	Versican	ACA AAG GGA GAG TGT CTG TG	CCC TGT AGT GAA ACA CAA CC
	CRABP1	GGC TTG CTC CTA CTT TCA G	CCA AAC TAT GGA GCA CTA GG
	NOGGIN	GAA CAC CCA GAC CCT ATC TT	CTC TAG CCC TTT GAT CTC G
	SOX2	ACA ACT CGG AGA TCA GCA	GCA GCG TGT ACT TAT CCT TC
	GAPDH	GAA GGT GAA GGT CGG AGT CAA	GCT CCT GGA AGA TGG TGA TG
